# Obesity-Related Metabolic Syndrome and Vascular Complications

**DOI:** 10.1155/2013/534056

**Published:** 2014-01-16

**Authors:** Manfredi Tesauro, Micaela Iantorno, Umberto Campia

**Affiliations:** ^1^Dipartimento di Medicina dei Sistemi, Università di Tor Vergata, 00133 Rome, Italy; ^2^Critical Care Medicine Department, National Institute of Health, Bethesda, MD 20852, USA; ^3^MedStar Heart Institute and MedStar Cardiovascular Research Network, Washington, DC 20009, USA

The epidemic of obesity, a condition defined as a body mass index (BMI) of 30.0 or more, has spread beyond the borders of high-income countries and is now dramatically on the rise also in low- and middle-income nations, particularly in urban settings [1]. One of the most concerning implications of this trend is the association between obesity and an increased risk of developing metabolic and cardiovascular disease in adults [2, 3] and in children [4–7]. Obesity, particularly when in a central body distribution, is frequently associated with the presence of the metabolic syndrome (MetS), a cluster of lipid abnormalities (i.e., elevated triglycerides and low HDL), hypertension, and impaired fasting glucose [8, 9]. Even if the MetS does not encompass risk factors that determine absolute risk (e.g., age, sex, cigarette smoking, and LDL cholesterol), it is associated with a two-fold higher risk of cardiovascular disease (CVD) at 5 to 10 years and a 5-fold increase in risk to develop type 2 diabetes mellitus (T2DM) [8]. However, the definition of MetS and the associated CVD risk have been validated for mostly white cohorts and they may not be valid for different populations [10].

Numerous pathophysiological mechanisms have been proposed to link central adiposity, MetS, T2DM, and cardiovascular risk. In particular, adipose tissue dysfunction, insulin resistance/hyperinsulinemia, sympathetic hyperactivity, and endothelial dysfunction have emerged as pivotal players [11]. Adipose tissue dysfunction is characterized by the presence of an inflammatory infiltrate, adipokine dysregulation, and release of inflammatory cytokines, which exert deleterious vascular effects in a systemic and paracrine fashion [12]. In turn, the abnormal vascular function may reduce skeletal muscle perfusion, thus worsening insulin sensitivity and establishing a self-perpetuating vascular-metabolic vicious cycle. However, our knowledge of the pathophysiology of vascular damage in obesity is still incomplete. A better mechanistic understanding could have important clinical implications, leading to the development of novel therapeutic strategies to reduce metabolic and cardiovascular risk in obesity. In this special issue, we have selected six papers investigating various aspects of the association between obesity-related metabolic syndrome and vascular complications.

To address the scarcity of studies that have examined the relation between obesity and cardiometabolic risk factors (CRFs) in population-based samples of children and adolescents, T. Lyngdoh et al. assessed the association between BMI, measured both at the age of 12–15 years (longitudinal analysis) and at the present time (cross sectional analysis), and several CRFs in 390 young adults aged 19-20 years in Seychelles (Indian Ocean, Africa). In this cohort, CRFs were strongly predicted by BMI levels at both ages. However, only BMI at age 19-20 years remained a strong predictor of CRFs in a regression model including both BMI at age 12–15 years and BMI at age 19-20 years. These data suggest that current BMI is a strong predictor of CRFs and indicate that weight control even at a later age may be effective in reducing CRFs in irrespective of past weight status.

Similar to other advanced countries, the prevalence of obesity in Turkey is higher than 30%. However, no population-specific cutoffs for waist circumference (WC) to use within the definition of MetS have been established. Therefore, A. Sonmez et al. investigated whether the World Health Organization's (WHO) sex specific WC cut-off points for the Caucasian adults are appropriate for the Turkish adult population. Using a sample of over 4,200 Turkish men and women, the authors determined that the most appropriate WC cut-off levels to diagnose MetS are 90 cm and 80 cm, respectively, and suggest that these cut-off values should be used in Turkey and in areas with large Turkish immigrant populations.

One of the well-recognized abnormalities in obesity is the hyperactivity of the sympathetic nervous system (SNS). As detailed in the review by M. P. Canale et al., the SNS is activated by multiple features present in obese individuals, including insulin resistance and adipokine unbalance. Importantly, chronic SNS overactivity contributes to a further decline of insulin sensitivity and creates a vicious circle that may contribute to the development of vascular damage and CVD. Importantly, preliminary evidence appears to suggest that renal denervation may be a potential therapy to treat sympathetic hyperactivity and its effects on blood pressure and metabolism in obese individuals.

A variable amount of adipose tissue can be found around most vessels. Until recently, the vascular effects of perivascular adipose tissue- (PVAD-) derived factors on vascular function had remained mostly unrecognized. The structural and functional changes observed in the PVAD of obese individuals and their impact on vascular function are reviewed in detail by M. S. Fernández-Alfonso et al. As noted by these authors, further research is necessary to better understand the role of PVAT in the pathogenesis of CVD in obesity and MetS.

Recent evidence suggests that inhibition of the renin angiotensin system (RAS) may reduce the risk of T2DM. To understand the potential mechanisms underlying this observation, Z. Zhang et al. investigated the effects of angiotensin II type 1 receptor (AT1R) knockdown on glucose-stimulated insulin secretion in isolated islets of db/db mice. Their results suggest that intraislet AT1R is a physiological regulator of insulin sensitivity in *β* cell and a potential therapeutic target for the prevention of T2DM.

Finally, extensive calcification of the vessel wall is one of the notable features of vascular remodeling in patients with T2DM and end stage renal disease (ESRD). Given the high prevalence of impaired glucose metabolism and diabetes in patients with ESRD, K. Janda et al. investigated the association between a number of clinical and metabolic parameters and radial artery calcification in patients with ESRD. In their study population, impaired fasting glucose and T2DM were significant predictors of vascular calcification, suggesting an independent contribution of abnormal glucose metabolism in the vascular remodeling of patients with ESRD.

These papers represent a very small sample of the research work in the field. However, we are confident that they will convey to the readers the breadth of the ongoing epidemiological, clinical, and laboratory efforts to unravel the pathogenetic mechanisms underlying the association between obesity, MetS, T2DM, and cardiovascular disease.

*Manfredi Tesauro* *Manfredi Tesauro* *Micaela Iantorno* *Micaela Iantorno* *Umberto Campia* *Umberto Campia*
